# The interplay of factors in metabolic syndrome: understanding its roots and complexity

**DOI:** 10.1186/s10020-024-01019-y

**Published:** 2024-12-27

**Authors:** Md. Sharifull Islam, Ping Wei, Md Suzauddula, Ishatur Nime, Farahnaaz Feroz, Mrityunjoy Acharjee, Fan Pan

**Affiliations:** 1 Center for Cancer Immunology, Institute of Biomedicine and Biotechnology, Shenzhen Institute of Advanced Technology, Chinese Academy of Sciences, Shenzhen, 518055 China; 2 Department of Microbiology, Stamford University Bangladesh, 51, Siddeswari Road, Dhaka, 1217 Bangladesh; 3 Department of Pediatric Otolaryngology Head and Neck Surgery, West China Second University Hospital, Sichuan University, Chengdu, China; 4 Department of Food Nutrition Dietetics and Health, Kansas State University, Manhattan, KS 66506 USA; 5 Key Laboratory of Environment Correlative Dietology, College of Food Science and Technology, Huazhong Agricultural University, Wuhan, Hubei China

**Keywords:** Metabolic syndrome, Epidemiology, Pathogenesis, Obesity, Insulin resistance, Dyslipidemia

## Abstract

Metabolic syndrome (MetS) is an indicator and diverse endocrine syndrome that combines different metabolic defects with clinical, physiological, biochemical, and metabolic factors. Obesity, visceral adiposity and abdominal obesity, dyslipidemia, insulin resistance (IR), elevated blood pressure, endothelial dysfunction, and acute or chronic inflammation are the risk factors associated with MetS. Abdominal obesity, a hallmark of MetS, highlights dysfunctional fat tissue and increased risk for cardiovascular disease and diabetes. Insulin, a vital peptide hormone, regulates glucose metabolism throughout the body. When cells become resistant to insulin’s effects, it disrupts various molecular pathways, leading to IR. This condition is linked to a range of disorders, including obesity, diabetes, fatty liver disease, cardiovascular disease, and polycystic ovary syndrome. Atherogenic dyslipidemia is characterized by three key factors: high levels of small, low-dense lipoprotein (LDL) particles and triglycerides, alongside low levels of high-density lipoprotein (HDL), the “good” cholesterol. Such a combination is a major player in MetS, where IR is a driving force. Atherogenic dyslipidemia contributes significantly to the development of atherosclerosis, which can lead to cardiovascular disease. On top of that, genetic alteration and lifestyle factors such as diet and exercise influence the complexity and progression of MetS. To enhance our understanding and consciousness, it is essential to understand the fundamental pathogenesis of MetS. This review highlights current advancements in MetS research including the involvement of gut microbiome, epigenetic regulation, and metabolomic profiling for early detection of Mets. In addition, this review emphasized the epidemiology and fundamental pathogenesis of MetS, various risk factors, and their preventive measures. The goal of this effort is to deepen understanding of MetS and encourage further research to develop effective strategies for preventing and managing complex metabolic diseases.

## Introduction

MetS poses a significant and growing challenge in the field of public health and clinical medicine worldwide. It encompasses a range of metabolic disorders, such as hyperglycemia, hypertension, visceral obesity, atherogenic dyslipidemia, endothelial dysfunction, and genetic susceptibility. These disorders arise as a result of factors such as urbanization, excessive calorie intake, sedentary lifestyles, and escalating rates of obesity. The concept of MetS dates back to the early twentieth century when Kylin, a Swedish physician, first reported the connection between high blood glucose levels (hyperglycemia), high blood pressure (hypertension), and inflammatory arthritis (Han et al. 2019; Kylin 1923). In 1965, Avogaro and Crepaldi reported a comparable syndrome characterized by hyperglycemia, hypertension, and obesity (Avogaro et al. 1965). The year was 1988 when Reaven introduced the groundbreaking idea of syndrome X (Hu et al. 2019; Reaven 1988). This term encompasses numerous risk factors that contribute to both diabetes and CVD, with IR being the main concept (Després, 2018). In 1989, Kaplan decided to give the syndrome a new name, namely, “The Deadly Quartet” (Kaplan 1989). This name was chosen because of its four main components: glucose intolerance, hypertriglyceridemia, hypertension, and upper-body obesity (Clearfield et al. 2014). The syndrome underwent another renaming in 1992 and was henceforth referred to as “the IR syndrome” (Al-Hamad & Raman 2017). Vague’s report revealed a significant link between visceral obesity and metabolic disorders responsible for causing diabetes and cardiovascular diseases (CVD) (Vague, 1996). In 2001, the National Cholesterol Education Program revised the definition of “MetS” (Cleeman 2001; Saif-Ali et al. 2020). Additionally, in April 2005, the International Diabetes Federation (IDF) introduced the term MetS (Group, 2005; Kaur 2014). The history surrounding MetS spans approximately one hundred years, and various researchers have made notable contributions to understanding this complex condition. The current definition encompasses multiple metabolic risk factors that augment an individual’s susceptibility to developing conditions such as diabetes, CVD, and other chronic diseases. Therefore, it becomes pivotal to prioritize efforts directed toward addressing and appropriately managing this syndrome in favor of public health outcomes.

The prevalence of MetS extensively varies around the world, depending on factors such as region, urban or rural environment, demographics (including age, sex, race, and ethnicity), and diagnostic criteria used (depending on the organization that is consulted) (Jamali et al. 2024; Khan et al. 2018). The diagnostic criteria differ among different organizations such as the World Health Organization (WHO), International Diabetes Federation (IDF), National Cholesterol Education Program (NCEP) ATP III, and other regional guidelines. Such segmentation leads to variability in MetS prevalence among populations. Although standards have fluctuated to some extent in particular components, in general, they encompass a synthesis of both fundamental and metabolic risk determinants (Grundy et al. 2005). The IDF estimated that approximately 20–25% of the adult population worldwide is affected by MetS (Jamali et al. 2024). Recent epidemiological evidence from the United States has revealed that the incidence of MetS has escalated from 27.6% to 32.3% over the last several decades (Tao et al. 2024). On top of that, in Europe, around 24.3% incidence of MetS has been documented according to the NCEP: ATP III criteria (Adjei et al. 2024). A recent investigation reported that in the Chinese population, the prevalence of metabolic syndrome (MetS) varies depending on the criteria used: 34.52% according to IDF (2006) criteria, 38.63% according to ATP (2005) criteria, 25.94% according to ATP (2001) criteria, 26.31% according to CDS (2004) criteria, and 21.57% according to WHO (1999) criteria (Ma et al. 2024). On top of that, the incidence of MetS in Africa was 32.4% but this occurrence fluctuates among demographics and could be as elevated as 50% or greater (Bowo-Ngandji et al. 2023; Charles-Davies & Ajayi 2023). For example in sub-Saharan Africa, the aggregated occurrence of the MetS was 21.01% according to NCEP/ATP III criteria and 23.42% according to IDF criteria (Asgedom et al. 2024). A study conducted on one of the most populated countries (i.e. India) found that the prevalence of MetS was around 4.83% based on NEPT/ATPIII criteria (Saurav et al. 2023). The increasing prevalence of MetS is a significant public health concern, necessitating focused research efforts to understand and mitigate its impact.

People with higher body weights are more likely to have MetS, with a prevalence of approximately 5% among regular-weight people, more than 20 among higher-weight people, and more than 50% among obese people, based on data from the National Health and Nutrition Examination Survey (Kim, 2022). According to the standards set by the NCEP ATP III in 2001, the prevalence of MetS varies significantly among different countries. It ranges from 8 to 43% in men, while in women, it varies between 7 and 56% (Alqahtani et al. 2022). The Framingham Heart Study reported that gaining ≥ 2.25 kg of weight over 16 years is linked to a 21% to 45% increase in the chance of having MetS (Palaniappan et al. 2004). The increasing incidence of obesity in adults may lead to even higher rates of MetS soon (Saklayen 2018).

The 1921 discovery of insulin opened doors for in-depth research on its effects (Li et al. 2022). The MetS refers to a constellation of conditions in which IR serves as a predominant characteristic of the pathology. This syndrome presents a significant risk for the onset of cardiovascular complications, while also adversely affecting the gut-liver axis, which encompasses the pancreas, primary liver, and colorectal-related immunity (Apaza et al. 2024). The modulation of immunometabolic responses through immunonutritional factors (IFs) has been recognized as a crucial determinant of the metabolic and immune health associated with the gut-liver axis. Immunonutritional factors derived from plant seeds have demonstrated in vitro and pre-clinical efficacy primarily in addressing various immunometabolic and inflammatory disorders (Apaza et al. 2024). The American Heart Association (AHA) has proposed the Cardiovascular-Kidney-Metabolic (CKM) syndrome, highlighting the interrelation of metabolic, renal, and CVD. There exists significant evidence linking the triglyceride glucose-body mass index (TyG-BMI) to CVD as a measure of IR. Nonetheless, it is unclear if this correlation applies to populations with CKM syndrome (W. Li et al. 2024a, b). However, CKM health denotes the interaction between metabolic risk factors, chronic kidney disease, and cardiovascular function, significantly influencing morbidity and mortality (Ndumele et al. 2023). This review examines the multiple risk factors associated with various metabolic diseases and their contribution to the development of MetS. We explore the global prevalence of MetS and its risk factors, analyze the effectiveness of different diagnostic criteria, investigate the impact of specific lifestyle interventions, and delve into the underlying mechanisms linking various risk factors to MetS development.

### The complex pathogenesis of metabolic syndrome

**Fig. 1:**
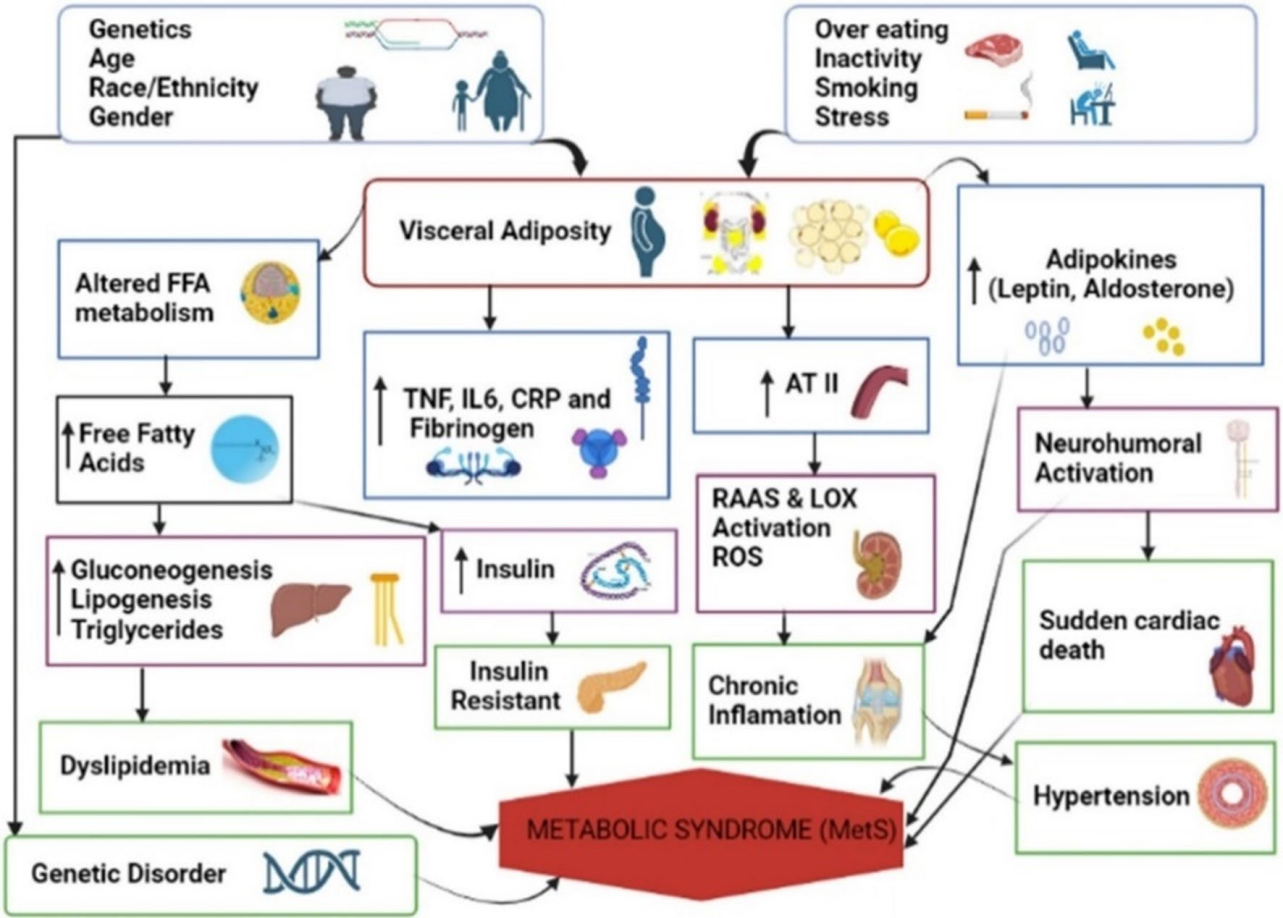
Schematic presentation of MetS. Multiple factors (genetics, age, lifestyle, overeating, inactivity, and smoking) lead to visceral adiposity, central to MetS. This adiposity triggers altered free fatty acid metabolism, promoting insulin resistance, dyslipidemia, and increased inflammation through markers like TNF, and IL-6. Additionally, adipokines (leptin, aldosterone) and the RAAS exacerbate chronic inflammation. These interconnected processes raise the risk of cardiovascular diseases like sudden cardiac death and hypertension, all key features of MetS. FFA: Free Fatty Acid, ATII: Angiotensin II, CRP: C-reactive protein, TNF: Tumor Necrosis Factor, IL-6: Interleukin 6, LOX: Lectin-like Oxidized, LDL: Low-Density Lipoprotein, ROS: Reactive Oxygen Species, RAAS; Renin Angiotensin Aldosterone System

The pathogenesis of MetS is thought to involve several complex pathways that have not been fully characterized. Medical and scientific experts became concerned about whether the various MetSs are linked by a single pathogenic pathway or fall under a mutual pathogenic process. The complicated interactions between different genetic and environmental factors, including overeating, smoking, stress, and physical activity, can influence the development of MetS (McCracken et al. 2018). Visceral adiposity is the crucial trigger that is correlated with most of the pathways involved in the development of MetS (Chait & den Hartigh, 2020). Chronic inflammation, IR, and neurohormonal activation play key roles in the complex syndrome known as MetS as it progresses. Numerous other factors, including genetic susceptibility, dyslipidemia, hypertension, and CVD, can have an impact on MetS (Fig. 1).

### Obesity, physical inactivity, and adipose tissue inflammation: key drivers of metabolic syndrome

An excessive buildup of body fat is the standard definition of obesity. Body weight has frequently been employed as an indirect predictor of adiposity because it might be difficult to measure adiposity precisely. Body mass index (BMI), which measures adiposity by dividing weight by height in kilos per square meter, was calculated. Cutoff points were established to identify overweight or obesity in adults (Nuttall 2015), and later, similar values were established for children and adolescents (Table 1). A sedentary lifestyle contributes to the development of risk factors for MetS, which can include hypertriglyceridemia, high levels of apolipoprotein B, low levels of high-density lipoprotein (HDL) cholesterol, small and dense low-density lipoprotein (LDL) and HDL particles, IR, inflammation, glucose intolerance, hyperinsulinemia, altered fibrosis, and endothelial dysfunction (Strasser 2013). These risk factors are strongly associated with CVD and T2D. Based on 1 week of accelerometer data collected from the National Health and Nutrition Examination Survey, it was found that most people spend the majority of their daily nonsleeping time either engaging in sedentary behavior (58%) or being inactive (39%), with only a small percentage (3%) engaging in regular exercise (Owen et al. 2010). The prevalence of inactivity is a major concern, particularly for individuals who do not regularly engage in physical exercise. Excessive sitting has been linked to a greater risk of developing MetS in the future (Booth et al. 2012). It has been reported that insufficient exercise, inactivity and extended periods of sitting can lead to obesity, which is connected with an elevated risk of developing diverse MetS and, ultimately, higher mortality rates (Hamilton et al. 2008).

**Table 1:** Categorize obesity based on BMI, weight range, and percentile range

Weight Range^1^	BMI^2^	Percentile Range^3^	Weight Status Category
< 124 lbs	< 18.5 kg/m^2^ (King 2007)	< 5 (Seibert et al. 2014)	Underweight
125–168 lbs	18.5–24.9 kg/m^2^ (King 2007)	5–85 (Seibert et al. 2014)	Healthy Weight
169–202 lbs	25–29.9 kg/m^2^ (King 2007)	85–95 (Seibert et al. 2014)	Overweight
≥ 203 lbs	30–39.9 kg/m^2^ (Uzogara 2016)	> 95 (Seibert et al. 2014)	Obesity (I-III)

^1^Based on male height of 5′ 9″. ^2^Adult age as ≥ 20 years and unit as kg/m^2^. ^3^Children and adolescents whose age between 2 to 19 years old

The increase in obesity rates resulting from increased consumption of high dietary energy density and decreased physical activity is referred to as an “obesity epidemic” (Mozaffarian 2022). Adipocytes, stromal cells, immune cells, and the endothelium are among several cell types that make up adipose tissue. Adipocyte hypertrophy and hyperplasia can be caused by these cells in response to rapid and strong changes in nutrient availability (Eckel-Mahan et al. 2020; Longo et al. 2019). Adipocyte growth increases when obesity worsens and can result in hypoxia by decreasing the blood supply to the surrounding tissue (Lee et al. 2010). Adipose tissue may experience necrosis and macrophage infiltration as a result of hypoxia or a lack of oxygen. This may result in the synthesis of numerous different metabolites, such as adipocytokines, free fatty acids (FAA), glycerol, tumor necrosis factor-alpha (TNFα), interleukin-6 (IL-6), C-reactive protein (CRP), proinflammatory mediators, and plasminogen activator inhibitor-1 (PAI-1) (Nieman et al. 2013). The resulting inflammation is confined to adipose tissue but can lead to systemic inflammation and the development of MetS (Ellulu et al. 2017). The regulation of many pathways, including oxidative stress, insulin sensitivity, energy metabolism, inflammatory reactions, and blood coagulation pathways, is regulated by adipocytokines that integrate endocrine, paracrine, and autocrine signals. In those who are affected, this causes the development of atherosclerosis, plaque rupture, and atherothrombosis (Fernández-Real & Ricart 2003; Kirichenko et al. 2022; Rehman & Akash, 2016). These findings demonstrated that adipose tissue serves as an endocrine organ that produces cytokines, which are intimately associated with the development of MetS, in addition to being in charge of storing and mobilizing lipids.

Observing that obese patients with diabetes and clinical signs and symptoms of CVD tended to have central body fat distribution in 1947, a French doctor named Vague coined the term “male-type” or “android obesity”. On the other hand, female body fat tends to accumulate in the lower gynoid region (Després, 2012). Compared with BMI, the waist-to-hip ratio (WHR) is significantly related to MetS and CVD (Pimenta et al. 2016; Robert Ross et al. 2020a, b), but it is controversial because it cannot differentiate between visceral and subcutaneous abdominal fat (Vatier et al. 2014). Nevertheless, waist circumference (WC) is significantly correlated with MetS and the development of CVD (Seyedhoseinpour et al. 2023). Specifically, a high BMI, high waist-to-height ratio and high WC ratio were found to increase the risk of hypertension, dyslipidemia, T2D, and CVD (Freedman et al. 2007; Seyedhoseinpour et al. 2023; Wildman et al. 2005). It has been suggested that overweight or obese patients with a high-risk body fat pattern, as indicated by simple anthropometric indices of total adiposity such as WC, are more likely to experience adverse health outcomes associated with MetS and CVD (Barreira et al. 2012).

### Waist circumference: a marker of visceral adiposity and cardiovascular risk

Visceral adiposity and abdominal obesity are the most prevalent forms of MetS, which has become a quantifiable medical condition (Després et al. 2008; Paley & Johnson 2018). However, it was found that there is a significant difference in visceral adiposity even with a given BMI, and WC was proposed as a marker of visceral adiposity (Wei et al. 2019). However, the correlation between WC and abdominal adiposity, particularly visceral or intra-abdominal obesity, is dependent on age and sex. These issues were not adequately addressed by the initial NCEP ATP III guidelines (Baek et al. 2020; Camhi et al. 2011). Interestingly, some cardiometabolic studies in cardiac patients have shown that WC is more strongly associated with total body fat mass and subcutaneous adiposity than with the quantity of visceral adiposity (Zhang et al. 2013). In 2020, Ross et al. reported that the average WCs of men and women with a BMI of 30 kg/m^2^ were 102 cm and 88 cm, respectively (Ross et al. 2020a, b). WC has been found to be linked to clinical outcomes, but defining the cutoff values is challenging, especially considering that women tend to have more subcutaneous fat and less visceral fat than men (Blaak 2001; Schorr et al. 2018). For certain ethnic groups, the IDF has been suggested to have lower WC cutoff values (He et al. 2017).

**Fig. 2:**
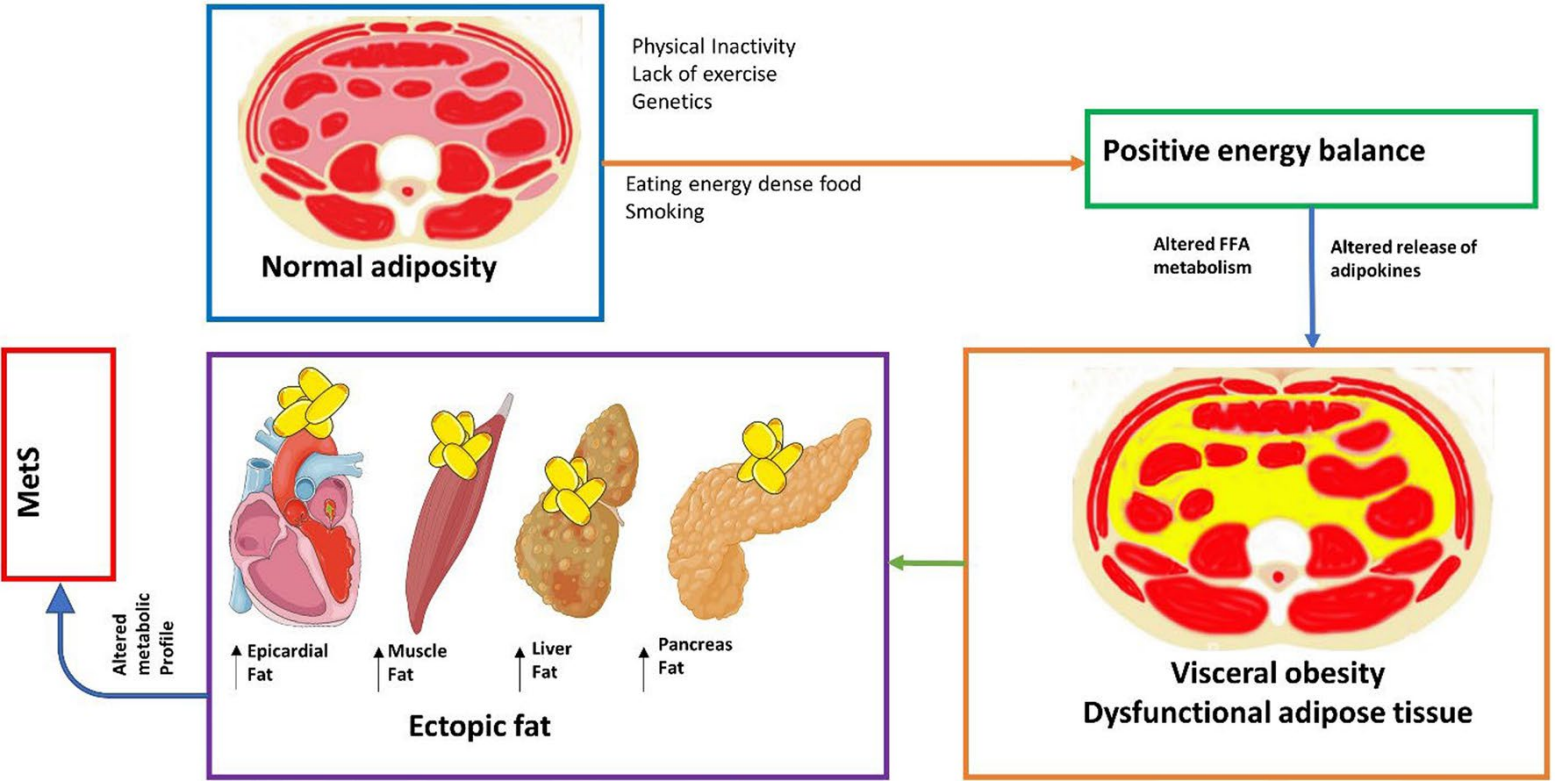
Pathophysiology of visceral obesity. Factors like inactivity, poor diet, and smoking cause positive energy balance, leading to visceral obesity and dysfunctional adipose tissue. These triggers altered fat metabolism and adipokine release, resulting in ectopic fat accumulation in organs like the heart, liver, and muscles, which ultimately drives MetS

However, these ethnicity-specific cutoffs do not always account for differences in visceral fat and clinical outcomes in other populations, so a proposed method for defining abdominal obesity-related risk in various populations is needed (Wang et al. 2012a, b). As a result, some researchers have suggested combining WC measurements with BMI to better assess obesity (Højgaard et al. 2008). Nevertheless, regardless of BMI, patients with a larger WC tend to have more abdominal fat than those with a smaller WC (Stevens et al. 2010). It is possible to identify a subgroup of individuals or patients with abdominal obesity by combining waist measurements with BMI and an understanding of the pathophysiology of visceral obesity, as illustrated in Fig. 2. Based on the current understanding of the pathophysiology of visceral obesity, Lemieux et al. proposed a clinical phenotype called “hypertriglyceridemic waist” over two decades ago, which identified individuals with excess visceral adiposity through the presence of hypertriglyceridemia and an enlarged WC (Lemieux et al. 2000). Since then, many studies have shown a strong connection between hypertriglyceridemic WC and cardiovascular risk, as well as other clinical phenotypes, such as MetS (Cai et al. 2022; de Cuevillas et al. 2021; LeBlanc et al. 2018). Convenient tools for screening individuals for increased visceral adiposity, related cardiometabolic abnormalities, and MetS include BMI, WC, and triglyceride measurements (Zhang et al. 2021).

### Insulin resistance and metabolic syndrome

**Fig. 3:**
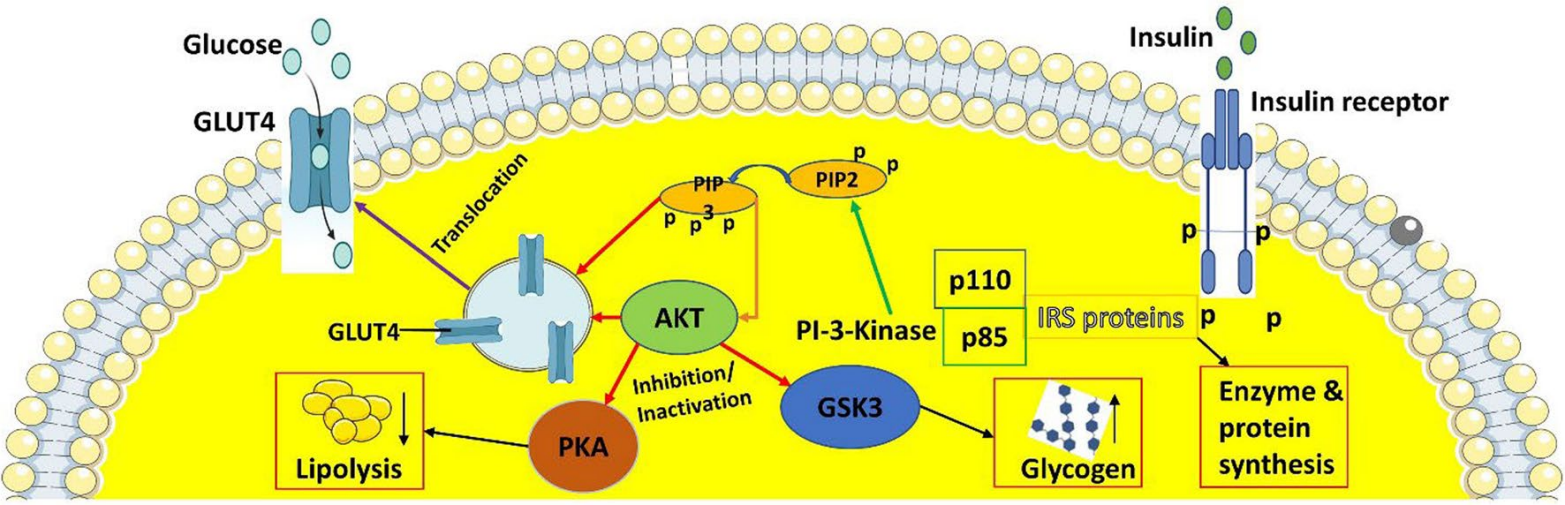
Schematic diagram of the mechanism action of insulin on an intracellular pathway. Insulin binds to the insulin receptor on the cell membrane, it triggers the phosphorylation (activation) of IRS (insulin receptor substrate) proteins. This, in turn, activates PI3-kinase, which converts PIP2 to PIP3, leading to the activation of AKT. Activated AKT promotes several downstream effects: it inhibits glycogen synthase kinase-3 (GSK3), which prevents glycogen breakdown and supports glycogen synthesis. AKT also facilitates the translocation of GLUT4 (a glucose transporter) to the cell membrane, enabling glucose uptake into the cell. Additionally, protein kinase A (PKA) is involved in the regulation of lipolysis, with insulin suppressing this process to reduce fat breakdown. The overall pathway enhances glucose uptake, glycogen storage, and reduces lipolysis, promoting energy storage and utilization. GLUT4: glucose transporter 4, PI-3: phosphoinositide-3, PIP2: phosphatidylinositol-4,5-bisphosphate, PIP3: phosphatidylinositol-3,4,5-triphosphate, PKA: protein kinase A

The anaerobic polypeptide hormone insulin is essential for controlling the metabolism of carbohydrates, proteins, and fats. One of its main functions is to target particular cells and encourage their uptake of glucose from the circulation of blood. This process is necessary to ensure that the body’s appropriate glucose levels are maintained and that cells are effectively utilizing glucose for energy synthesis and other metabolic functions. The function of insulin in the absorption and metabolism of glucose is illustrated in Fig. 3. Insulin is released by beta cells in the pancreas. Targeted cells, including those in skeletal muscle, the liver, and adipose tissue, contain glycoprotein receptors to which they bind to exert their effects (Dong et al. 2022). The insulin receptor is a heterotetrameric membrane protein composed of two extracellular α subunits that bind insulin and two β subunits that are located inside the cell membrane (Lee et al. 2014). Insulin receptor substrate (IRS) proteins and phosphoinositide 3-kinase (PI3K) cascades are activated by the insulin receptor to trigger many intracellular signaling pathways (Gorgisen et al. 2022). PI3K, the lipid kinase catalytic subunit, is usually converted from phosphatidylinositol-4,5-bisphosphate (PIP2) to phosphatidylinositol-3,4,5-triphosphate (PIP3) through phosphorylation reactions (Minami et al. 2021; Xie et al. 2019). Protein kinase B (AKT) is activated at the membrane by the triggered p110 catalytic subunit at the membrane (He et al. 2021). Glycogen synthase kinase 3 (GSK3) is then inhibited by phosphorylated, activated AKT, which promotes enhanced glycogen synthesis and supports the storage of glucose as glycogen (Papadopoli et al. 2021). Additionally, AKT also inhibits lipolysis by interfering with protein kinase A, the main enzyme responsible for the breakdown of adipose tissue (Cederquist et al. 2017; Ding et al. 2016).

An essential step in the insulin-dependent absorption of glucose by cells is the translocation of the glucose transporter protein (GLUT4) to the cell membrane. This procedure makes it easier for glucose to enter the cell and be used as an energy source (Fig. 3). Adenosine triphosphate (ATP) is a main component of the metabolism of cellular energy. It is secreted by the phosphorylation of glucose and is kept in the body as the energy-storing compound glycogen (Bonora et al. 2012; Zhang & Ma 2021). GLUT4 is a glucose transporter that is overexpressed in the brain, heart, adipose tissue, and skeletal muscle (Wang et al. 2020). When a person is fasting, their insulin levels drop, which causes GLUT4 to move from the cell membrane to intracellular storage spaces. However, in the absence of insulin, high levels of mutant PI3K and AKT may facilitate the translocation of GLUT4 to the cell membrane (Vishnu Prasad et al. 2010). Insulin plays a critical role in glucose metabolism by inhibiting gluconeogenesis and glycogenolysis while stimulating glucose storage. Insulin also promotes transcriptional gene regulation via lipogenesis and glycolysis pathways (Morral et al. 2007). The inhibition of transcription and function of hepatic gluconeogenic enzymes is mediated by Akt through the phosphorylation of the forkhead box class O-1 (FOXO1) transcription factor (Tsuchiya & Ogawa 2017). Other transcriptional regulators of gluconeogenesis inhibition include forkhead box class O-6, peroxisome proliferator-activated receptor γ coactivator 1-α (PGC1α), and cAMP response (D’Errico et al. 2011). Overall, the regulation of glucose metabolism is complex and involves multiple signaling pathways and transcriptional regulators. Insulin plays a key role in coordinating these processes and ensuring that glucose is used efficiently to meet the energy needs of the body.

Insulin-sensitive individuals tend to have a better metabolic profile characterized by normal body weight, non-visceral obesity, physical activity, and consumption of low-fat and low-energy foods (Garmes, 2024; Klöting et al. 2010; Lin et al. 2022; Rosenfalck et al. 2006; Schenk et al. 2008). Conversely, those who are insulin-resistant exhibit impaired insulin action and glucose metabolism, resulting in increased fasting glucose levels, hyperglycemia, increased glucose uptake by muscles, increased hepatic glucose production, and increased adipose tissue lipolysis (Gastaldelli 2022). During this process, intravenous administration of insulin can reduce insulin function, resulting in decreased glucose metabolism by insulin and decreased endogenous glucose production (Janssen, 2021). Molecular mechanisms, such as mitochondrial dysfunction, can cause metabolic disorders, leading to IR and T2D, which have recently become major causes of death (Rehman et al. 2020). It is thought that the pathophysiology of MetS is connected to IR, which is caused by excess fatty acids as a result of increased lipolysis (Gastaldelli et al. 2017). MetS, including T2D, polycystic ovary syndrome, nonalcoholic fatty liver disease (NAFLD), CVD, and cancer, such as breast, uterus, cervix, colon, esophagus, pancreas, kidney, and prostate cancer, can develop when pancreatic beta cells are unable to produce enough insulin for an extended period of time (Jensen et al. 1989). These diseases are typically associated with the metabolic effects of IR, leading to visceral adiposity, hypertension, hyperglycemia, endothelial dysfunction, a prothrombic state, hyperuricemia, elevated inflammatory markers, and dyslipidemia caused by different environmental and genetic factors (Szukiewicz, 2023). Recent genetic and biochemical investigations have shown that adipose tissue may develop IR due to the production of lipids and other circulating substances that increase IR in other organs (James & Stöckli 2021). Understanding the underlying mechanisms can aid in the development of more effective management and treatment approaches since IR is a major risk factor for diverse metabolic disorders.

IR can be caused by mutations in the insulin receptor gene or by blockage of the proximal components of the insulin signaling pathway, such as insulin receptor substrate (IRS) proteins or AKT (Kahn et al. 1976). AKT is an essential component of the insulin signaling pathway and is involved in the regulation of more than 100 different substrates, allowing insulin to perform many basic physiological metabolic functions. However, IR can result when the proximal insulin signaling pathway is phosphorylated as a result of cellular stresses. Intracellular stressors such as c-Jun N-terminal kinase (JNK), Ser/Thr kinases, new protein kinase Cs (PKCs), S6 kinase, and mechanistic target of rapamycin (mTOR) can activate the insulin signaling pathway. IR can result from the phosphorylation of IRS proteins or insulin receptors by certain stressors. Negative feedback mechanisms that block the insulin signaling pathway are key factors in the emergence of IR. These pathways may contribute to the chronic nature of IR, making it difficult to cure (Copps & White 2012). When it binds to the insulin receptor, insulin is a ligand-activated tyrosine kinase that activates downstream substrates through tyrosine phosphorylation. These binding initiates two parallel pathways: the mitogen-activated protein kinase (MAPK) pathway and the PI3K pathway. IR develops when the PI3K and AKT pathways are altered, leading to a change in the balance between the two parallel pathways. Inhibition of the PI3K and AKT pathways results in a reduction in the production of endothelial nitric oxide, which is responsible for endothelial dysfunction. Additionally, it decreases the translocation of GLUT4 (Izquierdo & Crujeiras 2019), which reduces skeletal muscle glucose absorption and increases fat glucose. Endothelin-1 (ET-1) continues to be produced, while the MAP kinase pathway remains unaltered, causing the emergence of vascular cell adhesion molecules and nonspecific stimulants in vascular muscle cells (Xu et al. 2023). As a result, IR consequently causes vascular irregularities that may lead to atherosclerosis. It is important to note that people with IR may not necessarily be obese; instead, they may have aberrant fat deposition, with upper body fat predominating. Compared to lower body fat, abdominal obesity is more strongly linked to IR and MetS (Semenkovich 2006). IR leads to a disruption in the balance between the PI3K and MAPK pathways, resulting in vascular abnormalities and metabolic dysfunction.

**Fig. 4:**
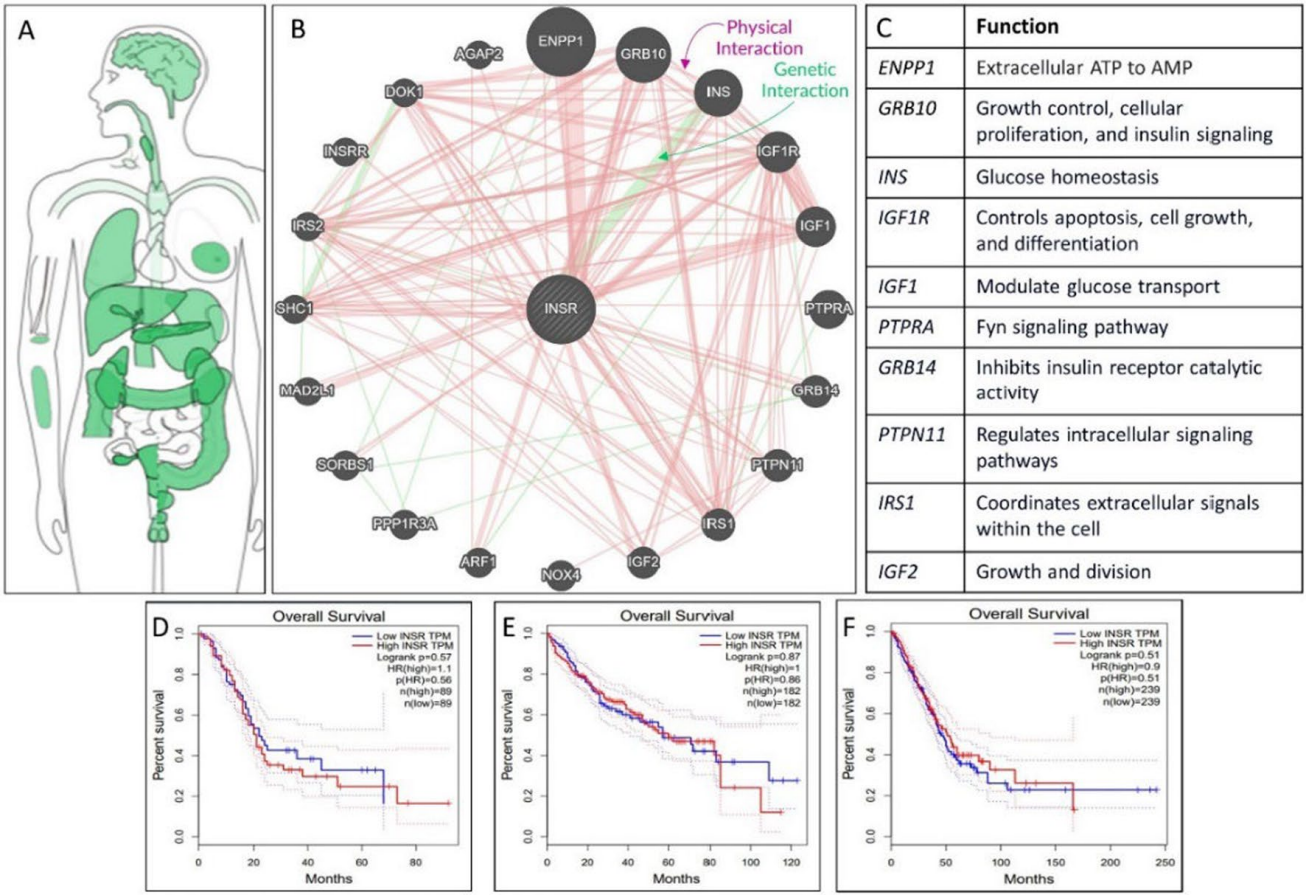
Domination of INSR on MetS. The median expression of INSR in normal samples (bodymap) (**a**), Gene Gene interaction and their function (Physical Interaction, Genetic Interaction) (**b**, **c**). Overall Survival with high and low INSR units as transcript per million in Pancreatic adenocarcinoma (**d**), Overall Survival with high and low INSR units as transcript per million in Liver hepatocellular carcinoma (**e**), Overall Survival with high and low INSR unit as transcript per million in Lung adenocarcinoma (**f**). The INSR network is highly interconnected with various signaling pathways and that its expression can significantly influence survival outcomes in diseases like cancer or metabolic disorders. The survival carbs and bodymap were made by GEPIA (http://gepia.cancer-pku.cn/index.html). Gene–gene interaction was made from GeneMANIA (https://genemania.org/)

In addition to these signaling pathways, several genes (Fig. 4b and c) related to MetS are correlated with the insulin receptor (INSR). Various mutations in the *INSR* gene contribute to IR (Wang et al. 2012a, b), and obesity is frequently linked to compromised INSR signaling, which commonly occurs before the development of overt T2D (Kushi et al. 2021; Skovsø et al. 2022). *INSR is* expressed throughout almost the entire human body, as shown in Fig. 4a (De Meyts 2016). As *INSR* is one of the central factors of MetS (Cornier et al. 2008; Jha et al. 2023), we analyzed overall survival with high and low INSR transcripts among three different chronic health conditions: pancreatic adenocarcinoma, liver hepatocellular carcinoma, and lung adenocarcinoma (Fig. 4d–f). Patients diagnosed with MetS are more likely to develop pancreatic cancer (Miyashita et al. 2024; Xia et al. 2020; Zhong et al. 2023). This association can be largely attributed to the presence of hypertension, hyperglycemia, and low levels of high-density lipoprotein cholesterol (HDL-c), which are known risk factors (Fig. 2) (Zhong et al. 2023). According to several epidemiological studies, individuals with MetS have an approximately 1.5–2 times greater risk of developing hepatocellular carcinoma (HCC) than those without MetS (Jinjuvadia et al. 2014). Moreover, the presence of MetS and its components, such as elevated WC, hyperglycemia, and low levels of HDL-c, are positively associated with the risk of lung cancer (Li et al. 2024a, b; Sin et al. 2020). In summary, IR could be considered a hallmark of MetS. It has been implicated in the development of lung cancer, liver cancer, and pancreatic cancer. The dysregulation of insulin signaling pathways observed in MetS may contribute to the increased risk of these cancers, highlighting the potential importance of targeting IR in prevention and treatment strategies.

### Dyslipidemia in metabolic syndrome

Dyslipidemia, a key component of MetS, encompasses conditions such as central obesity, insulin resistance (IR), and hypertension, which collectively heighten the risk of cardiovascular diseases (Burnett 2004). It is characterized by reduced HDL cholesterol levels and elevated levels of free fatty acids, triglycerides, apolipoprotein B, and very-low-density lipoprotein (VLDL) (González-Domínguez et al. 2023). IR, the primary underlying mechanism, disrupts normal lipid metabolism, leading to increased hepatic production of VLDL and impaired clearance of triglyceride-rich lipoproteins. These disruptions result in atherogenic dyslipidemia, marked by the accumulation of VLDL, small dense LDL, and low HDL-C, which accelerates atherosclerosis development. Chronic inflammation observed in MetS exacerbates these lipid abnormalities, further promoting endothelial dysfunction and plaque formation. Together, these metabolic and inflammatory disturbances significantly elevate the risk of coronary artery disease and other cardiovascular events in MetS patients.

Effective management of dyslipidemia in MetS is critical for reducing cardiovascular morbidity and mortality. Lifestyle modifications, including weight loss, increased physical activity, and dietary adjustments, are considered first-line interventions (Grundy 2005). An analysis of 11,549 dyslipidemic patients from the NHANES database revealed that lifestyle improvements significantly reduced all-cause mortality risk in individuals aged ≥ 65 years, regardless of lipid-lowering therapy, and similarly reduced mortality in younger patients (< 65 years) even without such therapies (Wang et al. 2023). Numerous studies have highlighted the positive effects of dietary components and bioactive elements on lipid profiles, contributing to dyslipidemia prevention and management (Rosa Cde et al. 2015). For instance, in a Spanish study of 211 untreated primary dyslipidemia patients, α-linolenic acid intake was inversely linked to atherosclerotic plaque risk in high-CVD-risk individuals (Sala-Vila et al. 2011). Additionally, increased hepatic uptake of free fatty acids (FFAs) in individuals with impaired glucose tolerance underscores the liver’s central role in dyslipidemic states (Ye et al. 2022). Notably, up to 80% of NAFLD patients experience dyslipidemia (Zhang & Lu 2015). Although no pharmacological agents are approved specifically for MetS, its risk factors are managed using therapies approved for cardiovascular and metabolic disorders. These include statins, PCSK9 inhibitors, antihypertensive medications, and novel glucagon-like peptide-1 receptor agonists (GLP-1 RAs) for type 2 diabetes and obesity treatment (Javor et al. 2024). The complexity of dyslipidemia in MetS necessitates individualized treatment strategies targeting lipid abnormalities and other components of the syndrome to mitigate long-term cardiovascular risks.

### Hypertension and metabolic syndrome

Hypertension and MetS are closely interrelated conditions that often coexist, collectively amplifying cardiovascular risk (Hezam et al. 2024). A thorough understanding of the molecular mechanisms linking these conditions is essential for identifying effective therapeutic targets. At the molecular level, insulin resistance (IR) plays a pivotal role in the development of both hypertension and MetS (Freeman et al. 2024; Ormazabal et al. 2018). Under normal conditions, insulin promotes vasodilation by stimulating nitric oxide (NO) production in endothelial cells via the PI3K-Akt pathway. However, in insulin-resistant states, this pathway is impaired, leading to reduced NO production and endothelial dysfunction, a key feature of hypertension. Simultaneously, IR activates the MAPK pathway, enhancing the activity of vasoconstrictors such as endothelin-1 (ET-1) and angiotensin II (Ang II), further elevating blood pressure (Muniyappa & Sowers 2013; Quesada et al. 2021; Sinha & Haque 2022; Zhou et al. 2014). In MetS, obesity-induced inflammation significantly contributes to the development of hypertension (Soleimani et al. 2023). Visceral fat is infiltrated by immune cells, such as macrophages, which release pro-inflammatory cytokines like TNF-α and IL-6 (Kawai et al. 2021). These cytokines exacerbate IR and stimulate the renin–angiotensin–aldosterone system (RAAS), increasing Ang II production. Ang II promotes vasoconstriction and sodium retention, driving blood pressure elevation (Hsueh & Wyne 2011). Chronic inflammation thus serves as a critical link between obesity, IR, and hypertension (Zatterale et al. 2019).

Dyslipidemia, another MetS component, also influences hypertension (Stanciu et al. 2023). Elevated free fatty acid (FFA) levels due to impaired lipid metabolism activate toll-like receptors (TLRs) on endothelial and immune cells, triggering inflammation and oxidative stress, which impair endothelial NO production (Ghosh et al. 2017; Goulopoulou et al. 2016). These processes worsen IR, increase vascular stiffness, and raise peripheral resistance, further contributing to hypertension. The kidneys play a crucial role in the interplay between MetS and hypertension (Luk et al. 2008). IR and obesity enhance renal sodium retention through sympathetic nervous system hyperactivation and RAAS upregulation (Thethi et al. 2012). This sodium retention, along with the kidneys’ diminished ability to excrete sodium properly in IR states, exacerbates volume expansion and sustains hypertension (Grillo et al. 2019).In conclusion, hypertension and MetS are intricately linked through molecular mechanisms involving IR, inflammation, dyslipidemia, and RAAS activation. These insights underscore the need for integrated therapeutic approaches that address both conditions simultaneously to mitigate long-term cardiovascular risks.

### Inflammation and metabolic syndrome

Inflammation is a core element of the pathophysiology of MetS, which includes obesity, insulin resistance (IR), dyslipidemia, and hypertension (Rochlani et al. 2017). Chronic low-grade inflammation serves as a critical link among these metabolic abnormalities, driving the progression of MetS and significantly increasing the risk of cardiovascular diseases and type 2 diabetes (Domingo et al. 2024). At the molecular level, visceral adipose tissue plays a pivotal role in the inflammatory processes associated with MetS. During obesity, adipocytes expand and become dysfunctional, resulting in hypoxia and tissue stress (Kang et al. 2023). This dysfunction recruits immune cells, particularly macrophages, into the adipose tissue, shifting their phenotype from an anti-inflammatory (M2) to a pro-inflammatory (M1) state. These macrophages release pro-inflammatory cytokines, including tumor necrosis factor-alpha (TNF-α), interleukin-6 (IL-6), and interleukin-1β (IL-1β), which propagate systemic inflammation and aggravate IR, a hallmark feature of MetS (Man et al. 2022; Strizova et al. 2023; Villarroya et al. 2018).

Dysfunctional adipocytes also release excessive free fatty acids (FFAs), which further amplify inflammation. Circulating FFAs activate toll-like receptors (TLRs), particularly TLR4, on immune and endothelial cells, triggering the nuclear factor kappa-light-chain-enhancer of activated B cells (NF-κB) signaling pathway (Renovato-Martins et al. 2020). This pathway stimulates the production of additional pro-inflammatory cytokines, perpetuating inflammation and promoting IR. Adipokines secreted by adipose tissue, such as leptin and adiponectin, further modulate inflammation (Clemente-Suárez et al. 2023). Leptin, elevated in obesity, enhances inflammation by activating macrophages and T cells, while adiponectin, an anti-inflammatory adipokine, is reduced in obesity (Pessin & Kwon, 2013). The imbalance between these adipokines in MetS intensifies the inflammatory response. In summary, chronic inflammation drives the onset and progression of MetS. Inflammatory processes within adipose tissue cause systemic effects, including IR, endothelial dysfunction, and hypertension, collectively heightening the risk of metabolic and cardiovascular diseases. Addressing inflammation and restoring a balanced inflammatory profile in individuals with MetS could serve as a critical therapeutic strategy to mitigate its associated health risks.

### Genetics and metabolic syndrome

The role of genetics in MetS is increasingly acknowledged, with both genetic predispositions and environmental factors contributing to its development (Fanning & O’Shea 2018). The genetic basis of MetS involves numerous genes that regulate key metabolic pathways, including lipid metabolism, insulin signaling, adipogenesis, and inflammation. Obesity, a central component of MetS, is strongly influenced by genetic factors. Polymorphisms in the FTO (fat mass and obesity-associated) gene, for instance, are associated with increased body mass index (BMI) and obesity. These variants affect appetite regulation and energy expenditure, promoting fat accumulation and adiposity in individuals carrying risk alleles (Song et al. 2023). Similarly, variants in the MC4R (melanocortin-4 receptor) gene, which regulates energy balance and appetite, are linked to increased fat mass and weight gain (Aykut et al. 2020).

Genetic regulation of lipid metabolism also plays a significant role in MetS. Variants in the APOA5 and LPL (lipoprotein lipase) genes are implicated in dyslipidemia, a hallmark feature of MetS. Polymorphisms in APOA5 contribute to hypertriglyceridemia by impairing the breakdown and clearance of triglyceride-rich lipoproteins, while mutations in LPL hinder lipolysis, leading to elevated triglycerides and low-density lipoproteins (LDL) (Hegele 2016; Lin et al. 2016; Smith et al. 2010). In addition, genes involved in inflammation, such as TNF (tumor necrosis factor-alpha) and IL6 (interleukin-6), are linked to elevated inflammatory responses in MetS, exacerbating its progression (Ting et al. 2020). Overall, genetic predispositions, in combination with lifestyle factors like diet and physical activity, contribute to the complexity and progression of MetS. Understanding the genetic architecture of MetS can aid in identifying at-risk individuals and developing personalized therapeutic strategies that target the underlying genetic and molecular mechanisms driving this multifaceted condition.

### The diagnostic criteria and management of MetS

The diagnostic criteria for MetS have evolved over the years, reflecting advancements in scientific understanding. Numerous international organizations and expert panels have endeavored to integrate the various parameters utilized in defining MetS (Kassi et al. 2011). Currently, the most common definitions come from NCEP: ATP III and IDF. They focus on waist size as a way to tell if someone has too much fat around their middle. On the other hand, the AACE, WHO, and EGIR definitions mostly look at how the body handles insulin, a hormone related to blood sugar (Kassi et al. 2011). MetS is diagnosed when an individual presents with three or more of the following criteria (Huang 2009): abdominal obesity (WC ≥ 94 cm in men or ≥ 80 cm in women), elevated triglycerides (≥ 150 mg/dL or specific treatment for elevated triglycerides), reduced HDL cholesterol (< 40 mg/dL in men or < 50 mg/dL in women), elevated blood pressure (≥ 130/85 mmHg or use of antihypertensive medication), and elevated fasting glucose (≥ 100 mg/dL or known type 2 diabetes) (Alberti et al. 2009; Sharma et al. 2020) (Table 2). Although a previous study fixed a cutoff value for WC, the latest literature stated that it should be determined based on population and country specifications (Alberti et al. 2009).

**Table 2:** Overall management approach of MetS

Component	Intervention	Details	Reference
Assessment	Framingham Risk Score	High Risk (10-year risk ≥ 20%) Moderately High Risk (10-year risk 10% to 20%) Lower to Moderate Risk (10-year risk ≤ 10%) Diagnosis of MetS using diagnostic criteria (Table 2)	Wilson et al. (1998)
Exercise	Physical Activity	Daily moderate-intensity activity for at least 30 min, most days of the week Encourage use of pedometer with goal of > 10,000 steps/day	Musto et al. (2010), Strasser, (2013)
Diabetes Prevention/Diet	Lifestyle Modification	Intensive lifestyle modification as primary therapy Weight reduction of 5–10% over four to 6 months Sodium intake < 65–100 mmol/day, aim for 90–120 mmol/day potassium Mediterranean diet and Dietary Approaches to Stop Hypertension (DASH) diet recommendations Consider low glycemic index foods, unrefined carbohydrates, soluble fibers, and balanced protein/fat intake Metformin as second-line for delaying onset of T2DM Thiazolidinediones and alpha-glucosidase inhibitors as third-line options	Bhoite et al. (2023), Hossain et al. (2018), National High Blood Pressure Education, (2004), Rashid et al. (2019), Vulin et al. (2022)
Blood Pressure Control	Treatment Initiation	Categorical Hypertension (BP ≥ 140/ ≥ 90 mm Hg) Established Diabetes malate’s (≥ 130/ ≥ 80 mm Hg) ACEIs/ARBs as first-line agents may decrease occurrence of diabetes. Could be affect glucose tolerance because of Beta-blockers and thiazides but paybacks in reaching blood pressure goal and reduce CVD risk	Mongkolsomlit et al. (2012), Nsutebu et al. (2020)
Cholesterol	Lipid Management	1st aim: LDL 2nd aim: non-HDL 3rd aim: HDL 4th aim: CRP	Doyle et al. (2014), Fiévet & Staels, (2009), Huijgen et al. (2012), Kandelouei et al. (2022)
	Therapeutic	Statin therapy to achieve LDL-C targets based on risk categories Consider niacin and/or fibrates once statin maximized Fibrates may be considered, especially for combined hypertriglyceridemia/low HDL-C Further LDL-C reduction with statin therapy, consider niacin for low HDL-C Statin therapy for those with high sensitivity CRP (hsCRP) ≥ 3 mg/dL	Doyle et al. (2014), Fiévet & Staels, (2009), Huijgen et al. (2012), Kandelouei et al. (2022)
Aspirin	Risk Stratification	Most Risk: Aspirin is advantageous Top-Intermediate Risk (10–20%): Aspirin likely advantageous Moderate Risk (6–10%): Individual clinical judgment, bearing in mind of gender and blood loss risk Less Risk (< 6%): Risk of hemorrhage outweighs benefits	Masson et al. (2022)

Identification and management of MetS patients is crucial to implement treatments that reduce their risk of subsequent diseases. Effective preventive strategies involve lifestyle modifications such as weight loss, diet changes, and exercise, complemented by appropriate pharmacological interventions to target specific risk factors. When Non-pharmacological treatments (NPT) measures failed or insufficient, pharmacological treatments should be considered (Ursa Herguedas, 2021). Clinical management of MetS is challenging due to the absence of a recognized method to address the entire syndrome, typically focusing on individual components, particularly those amenable to drug treatments (Spinler, 2006). The therapeutic objectives for MetS encompass reducing both short-term and lifetime cardiovascular risk, with the syndrome itself indicating a heightened lifetime risk (Grundy et al. 2005). A practical method for assessing short-term CHD risk in MetS patients without atherosclerotic cardiovascular disease (ASCVD) or diabetes involves utilizing the Framingham algorithm to estimate a 10-year CHD risk (Adil et al. 2023). This algorithm, incorporating factors like smoking, blood pressure, cholesterol levels, HDL-C, and age, categorizes patients into high risk (≥ 20%), moderately high risk (10–20%), or lower to moderate risk (≤ 10%) groups. Patients with ASCVD or diabetes are inherently considered high risk without requiring Framingham risk scoring (Duttagupta et al. 2022). On the other hand, lifestyle modification treatment involves a multidisciplinary approach, incorporating various healthcare professionals such as physicians, dieticians, exercise physiologists, psychologists, and health educators. While it may not achieve as significant reductions in individual risk factors as specific drugs might, lifestyle therapy offers benefits by moderately reducing overall metabolic risk factors (Figorilli et al. 2022).

Whereas four main approaches are recommended for weight reduction such as calorie restriction, increased physical activity, behavioral modification, (Wadden et al. 2020) and FDA-approved weight-reducing drugs. The recommended strategy to reduce weight is to target of 10% reduction in body weight within the first 6 months to a year, with continued weight loss until reaching a BMI below 25. Even modest weight loss of 5–10% can lead to significant improvements in various health markers such as triglycerides, HDL cholesterol, blood pressure, blood glucose, insulin levels, and hemoglobin A1c (Wadden et al. 2020). Regular exercise is crucial for abdominal fat loss and preventing weight regain. Combining calorie restriction, exercise, and behavioral changes can lead to meaningful weight loss and improvement in conditions related to obesity and MetS, including diabetes prevention (Shakoor et al. 2021; Wadden et al. 2020). Moreover, Current physical activity guidelines recommend 30 min of moderate-intensity exercise daily, with a preference for 60 min of brisk walking supplemented by other activities. High-risk patients should undergo clinical assessment before starting an exercise regimen, with medical supervision for some. Exercise plans should consider individual barriers and be gradually increased in intensity and duration. Brisk walking is encouraged, with patients instructed to gradually increase steps to 10,000–12,000 per day 189 (Musto et al. 2010; Strasser 2013; Yang 2019). Multiple short bouts of exercise are beneficial, and even 10-min sessions contribute to weekly energy expenditure. Regular exercise improves insulin sensitivity, reduces lipid levels, and lowers the risk of MetS and cardiovascular disease (Caro et al. 2013). Sedentary individuals should aim for at least 150 min of physical activity per week to gain health benefits. However, an effective long-term weight loss strategies involve reduced-energy diets with a moderate reduction of 500–1000 cal per day (Kim 2021). Referral to a registered dietician can ensure proper implementation and micronutrient intake while cutting calories. Mediterranean-style diets are associated with lower incidence of MetS and improved quality of life (Salas-Salvadó et al. 2016). The Dietary Approaches to Stop Hypertension (DASH) diet, along with lifestyle changes, improves metabolic parameters, especially blood pressure (Challa et al. 2024; Filippou et al. 2020). Maintaining a diet with 25–35% of calories from fat is recommended (Iacono et al. 2009), while protein intake should be 10–35% of total calorie intake, with exceptions for certain medical conditions. Structured meal plans, grocery lists, and meal replacements can enhance dietary adherence and aid weight loss. Sodium restriction and increased potassium intake are recommended for blood pressure control. Monounsaturated and polyunsaturated fatty acids, viscous fibers, and low glycemic index foods are beneficial for improving lipid profiles and glycemic control in individuals with or at risk of MetS (Siri et al. 2023).

### The current advancement in MetS research

#### Gut microbiota in MetS

The gut microbiota’s role in MetS has gained considerable attention due to its profound impact on metabolic health. Emerging evidence underscores the intricate interactions between gut microbiota, inflammation, and metabolic processes, highlighting how alterations in microbial composition contribute to the development and progression of MetS. For instance, individuals with MetS exhibit significantly reduced microbial diversity compared to the general population. Of the 930 operational taxonomic units analyzed in one study, 529—representing approximately 80% of total abundance—were strongly associated with MetS or its components (He et al. 2018). Similarly, a study using 16S rRNA metagenomic sequencing to characterize the gut microbiota of 111 MetS patients from the CORE-Thailand registry found that gut microbiota profiling may serve as a valuable tool for assessing and refining therapeutic strategies (Wutthi-in et al. 2020). These correlations between microbial species abundance and host characteristics often vary depending on the specific microbial community, emphasizing the need for targeted approaches (Wutthi-in et al. 2020).

Short-chain fatty acids (SCFAs), such as acetate and butyrate, produced by gut microbiota, play a vital role in regulating glucose and lipid metabolism, inflammatory pathways, and intestinal barrier integrity—key factors in MetS pathophysiology (Olalekan et al. 2024). Consequently, therapeutic interventions aimed at modulating gut microbiota composition and enhancing SCFA production have potential as novel approaches for managing MetS. Targeting these microbial pathways could provide effective strategies for mitigating the metabolic and inflammatory disturbances associated with this condition..

#### Epigenetic regulation in MetS

Epigenetic regulation represents a promising frontier in MetS research, as numerous genes are implicated in its pathogenesis (Silva-Ochoa et al. 2023). Epigenetic mechanisms, including DNA methylation, histone modifications, and non-coding RNAs (e.g., microRNAs), modulate gene expression without altering the DNA sequence. These mechanisms are highly sensitive to environmental factors such as diet, lifestyle, and metabolic status, playing a critical role in the development and progression of MetS. A study revealed no significant changes in ATP5E CpG methylation between MetS and non-MetS groups; however, COX6C showed markedly reduced methylation at CpG sites 2 and 4 in the MetS group (both *p* < 0.001). Similarly, RPL9 methylation levels were significantly reduced at positions 2 (*p* < 0.05), 3 (*p* < 0.0001), and 4 (*p* < 0.01) in MetS individuals (Urashima et al. 2021). Another investigation demonstrated a correlation between MetS components and accelerated epigenetic aging, with GrimAgeAA as an indicator. Increased waist circumference, triglycerides (0.218, *p* = 2.6E-4), and fasting glucose (0.163, *p* = 0.027), alongside decreased HDL cholesterol (− 0.231, *p* = 0.001), were strongly associated with higher GrimAgeAA, highlighting the role of epigenetic aging in MetS (Föhr et al. 2024). Understanding epigenetic mechanisms may enable advancements in diagnosing and managing metabolic disorders through epigenetic biomarkers, pharmaceuticals, and modification techniques (Wu et al. 2023). Emerging research into the interplay between the metabolome and epigenome may reveal novel biomarkers for disease prediction and therapeutic targets, particularly in the context of age- and sex-related epigenetic drift. Moreover, certain drugs, such as sitagliptin, show potential in preserving vascular function in MetS via epigenetic regulation. These findings emphasize that epigenetics offers critical insights into MetS complexity while paving the way for innovative therapeutic approaches.

#### Metabolomic profiling for early detection of metabolic syndrome

Metabolomic profiling offers a promising method for the early detection of MetS by identifying metabolic biomarkers that differentiate individuals with MetS from healthy or obese individuals without the syndrome. A comparative study involving populations from the United States and Japan highlighted key biochemical pathways implicated in MetS, including branched-chain amino acid metabolism, glutathione synthesis, aromatic amino acid metabolism, gluconeogenesis, and the tricarboxylic acid cycle. These findings underscored the significance of altered amino acid metabolism as a hallmark of MetS (Roberts et al. 2020). In another study, 56 metabolites specific to MetS were identified and replicated, with 13 showing positive associations (e.g., Valine, Leucine/Isoleucine, Phenylalanine, and Tyrosine) and 43 showing negative associations (e.g., Glycine, Serine, and 40 lipid species)(Shi et al. 2023). Notably, lysoPC a C18:2 was inversely correlated with MetS and all five of its components, while two acylcarnitines (C0 and C3) were positively associated with abdominal obesity, hypertriglyceridemia, and low HDL cholesterol levels (Shi et al. 2023). Similarly, a separate investigation identified five metabolites—LysoPC (14:0), LysoPC (15:0), propionyl carnitine, phenylalanine, and docosapentaenoic acid (DPA)—to develop a metabolite risk score (MRS), which demonstrated a dose–response relationship with MetS and related metabolic abnormalities (Wu et al. 2021). These findings demonstrate the potential of metabolomics to uncover disease-specific metabolic profiles, enabling early diagnosis and monitoring of MetS progression. By integrating metabolomic insights into clinical practice, personalized healthcare strategies could be developed to intervene early and more effectively manage MetS.

## Discussion

MetS is a collection of metabolic abnormalities, including physiological, biochemical, clinical, and metabolic factors that increase the risk of atherosclerotic cardiovascular disease (ACVD), T2D, and other diseases that cause mortality. Several diverse factors contribute to the development of MetS, including IR, visceral adiposity, dyslipidemia, genetic susceptibility, endothelial dysfunction, a hypercoagulable state, elevated blood pressure, and acute and chronic inflammation. This complex multifaceted endocrine syndrome is recognized as a risk factor for both nonatherosclerotic and atherosclerotic cardiovascular disease worldwide. Ectopic fat accumulation is a critical factor that contributes to the development of MetS and is associated with a proinflammatory state that may lead to the development of different types of diseases, including NAFLD, T2D, and CVD. Although individuals with acceptable LDL-C levels may be considered to have a low risk for CVD, those who are overweight or obese and demonstrate a dysmetabolic state or inflammation are still at increased risk for CVD. The World Health Organization (WHO) recently reported that MetS contributes significantly to mortality worldwide, with physical inactivity, high BMI, exposure to ambient particulate matter pollution, and air pollution from household solid fuels being the four leading causes. Thus, it is crucial to recognize and address MetS as a global public health concern to reduce morbidity and mortality linked with CVD, T2D, and other related diseases.

Obesity is a complex medical condition characterized by excessive body fat accumulation, particularly in the visceral adipose tissue or liver. Reduced insulin sensitivity in the body is the main determinant of obesity. Weight gain reduces insulin sensitivity, whereas weight loss increases it. Obesity and low insulin levels are determinants of MetS, which is associated with a positive metabolic phenotype. The cardiometabolic risks associated with overweight or obesity, including visceral adiposity, liver fat, and other ectopic fats (e.g., heart and pancreas), contribute to the risk of various cardiovascular diseases. An increase in WC can be a prognostic factor for elevated levels of abdominal fat at any given BMI. High WC can indicate excess visceral adiposity, leading to increased triglyceride levels. Community cardiologists and family physicians can identify overweight or obese patients with extra visceral adiposity and ectopic fat using markers such as WC and triglyceride levels. Obesity is the major fundamental risk factor for ASCVD and is linked to many ASCVD risk factors. Obesity is also a risk factor for T2D, which is itself a risk factor for cardiovascular disease. Although the mechanisms linking obesity, diabetes, and ASCVD are not yet fully understood, many metabolic pathways are activated during obesity, and these pathways are associated with the secretion of numerous potential risk factors. It is challenging to distinguish which risk factors are more significant and which are less important. However, there is high heterogeneity in the incidence of MetS due to the different factors that regulate IR. For example, lipoprotein metabolism may be regulated by genetic factors or diet, while blood pressure may be regulated by physical activity or dietary factors. T2D may develop in individuals with obesity or IR (Fahed et al. 2022). Aging is often associated with fat gain, loss of muscle mass, and accumulation of fat in the abdomen, which can increase IR and the risk of T2D (Al-Sofiani et al. 2019). Aging is also associated with specific faults in fatty acid oxidation in muscle, which further increases IR. Mild hypercortisolism is implicated in the development of abdominal obesity, while hyperandrogenism is associated with IR in women and can cause polycystic ovary disease. An overall management approach is given in Table 3 derived from existed literatures.

**Table 3:** Diagnostic criteria of the MetS

Dobrowolski et al	Criteria Dobrowolski et al. (2022)	
Waist Circumference	Man ≥ 102 cm, Women ≥ 88 cm	Waist circumference or BMI plus two or three criteria
Body Mass Index	≥ 30 kg/m^2^	
Fasting Glucose	≥ 100 mg/dl or ≥ 140 mg/dl after 120 min in oral glucose tolerance test	
Hemoglobin A	≥ 5.7%	
Non-High-Density Lipoprotein Cholesterol	≥ 130 mg/dl	
Blood pressure	Systolic ≥ 130 and/or Diastolic ≥ 85 mm Hg (in-office measurement), Diastolic ≥ 80 mm Hg (home measurement)	
On-going medication	On glucose-lowering drugs On Lipid-lowering drug On anti-hypertensive drug	
IDF	Criteria Alberti et al. (2006)	
Waist Circumference	Europids: man ≥ 94 cm, ≥ 80 cm. South Asians: Male ≥ 90 cm, Female ≥ 80 cm. Chinese: Male ≥ 90 cm, Female ≥ 80 cm. Japanese: Male ≥ 85 cm, women ≥ 90 cm	An individual is deemed to have MetS if he or she has central obesity plus any two of four factors
Triglycerides	≥ 1.7 mmol/l (150 mg/dl) or specific treatment for this lipid abnormality	
High-Density Lipoprotein cholesterol	< 1.03 mmol/l (40 mg/dl) in males, < 1.29 mmol/l (50 mg/dl) in females, or specific treatment for this lipid abnormality	
Blood pressure	Systolic: ≥ 130 mmHg, or Diastolic: ≥ 85 mmHg or treatment of previously diagnosed hypertension	
Fasting plasma glucose	≥ 5.6 mmol/l (100 mg/dl), or previously diagnosed Type 2 diabetes,	
NCEP ATP III	Criteria Cleeman, (2001)	
Waist Circumference	Man ≥ 102 cm Women ≥ 88 cm	Three or more of the five risk factors
High-density Lipoprotein cholesterol	Man < 1.04 mmol/L, Women < 1.30 mmol/L	
Blood pressure	Systolic ≥ 130, Diastolic ⩾85 mm Hg	
Fasting Glucose	≥ 110 mg/dL	
Triglyceride	≥ 1.70 mmol/L	
WHO	Criteria Comment on the provisional report from the WHO consultation, (1999), Takamiya et al. (2004)	
Waist Circumference	≥ 94 cm in men, ≥ 80 cm in women	Glucose intolerance, IGT or diabetes and/or IR together with two or more factors
fasting plasma glucose	≥ 6.1 mmol/l (nondiabetic)	
Blood pressure	Systolic ≥ 140 mmHg, Diastolic ≥ 90 mmHg or treated for hypertension	
Triglycerides	> 2.0 mmol/l or treated for dyslipidemia	
High-Density Lipoprotein cholesterol	< 1.0 mmol/l or treated for dyslipidemia	
AACE	Criteria Grundy et al. (2004)	
Overweight/obesity	BMI ≥ 25 kg/m2	
Triglycerides	≥ 1.70 mmol/L	
High-Density Lipoprotein cholesterol	Men < 1.04 mmol/L, Women < 1.30 mmol/L	
blood pressure	≥ 130/85 mm Hg	
Fasting glucose	6.1 to 6.9 mmol/L	
2-Hour post-glucose challenge	> 7.8 mmol/L	
Other risk factors	Family history of type 2 diabetes, hypertension, or CVD. Polycystic ovary syndrome. Sedentary lifestyle Advancing age Ethnic groups having high risk for type 2 diabetes or CVD	
EGIR	Criteria Balkau & Charles, (1999)	
Fasting plasma glucose	≥ 6.1 mmol/l (110 mg/dl) but non-diabetic	IR (defined as hyperinsulinemia—top 25% of fasting insulin values among the non-diabetic population). Plus two other factors
Blood pressure	≥ 140/90 mmHg or treatment	
Triglycerides	> 2.0 mmol/l (178 mg/dl) or or treatment	
High-Density Lipoprotein cholesterol	< 1.0 mmol/l (39 mg/dl)	
Waist Circumference	Men ≥ 94 cm, women ≥ 80 cm	

Dyslipidemia refers to elevated levels of various lipids in the bloodstream, and it is associated with several factors, such as obesity, a high-fat diet, smoking, and a sedentary lifestyle. Dyslipidemia is a risk factor for peripheral vascular disease, and the cellular mechanisms underlying atherosclerosis primarily cause dyslipidemia. Cholesterol can produce many components that contribute to the initiation, progression, and development of atherosclerotic plaques. The most well-known of these components are the protective effects of HDL and the pro-atherosclerotic effects of LDL. Other cholesterol components, such as TG and modified lipid proteins, also play important roles in vascular disease. When LDL becomes oxidized, it can contribute to oxidative stress and inflammation, which in turn can promote the development of vascular disease. Dyslipidemia is significantly linked to overweight and obesity, which increase the risk of hypertension. It is essential to focus on the early prevention and control of hypertension, including lifestyle interventions and subsequent health guidance, as soon as possible to reduce hypertension in high-risk groups.

### Limitations and future trends

While there have been some studies on the impact of peripheral stimuli and environmental factors on obesity, more research is needed to determine whether certain types of obesity are more susceptible to these factors and whether they contribute to a cytokine response that accelerates cardiovascular risk. To target weight loss effectively, it is important to focus on reducing WC and circulating triglyceride levels, which are key indicators of abdominal obesity and ectopic fat development, both of which can lead to cardiorespiratory issues. To gain a better understanding of the underlying mechanisms behind IR, future studies should explore variations in genes that regulate different cellular processes across various organs. Rather than relying on isolated components, a more systematic approach involving multiple omics methods is needed to elucidate the molecular landscape. There is also a need for research that can shed light on the mechanisms underlying chronic inflammation in individuals with obesity and the role of tissue immune crosstalk in MetS. Additionally, more research is needed to explore how to reduce the antagonistic side effects caused by mTOR signal blockade, the impact of different PI3K inhibitors on the transport and differentiation of pathogenic T cells, and the downstream effectors of PI3K that may be involved in metabolic diseases. Identifying the signaling pathways involved in T cells will be a significant challenge for future therapeutic modalities. It will be necessary to study Treg cells across different signaling pathways to facilitate the development of immunotherapies for MetS. Future research in the field of MetS needs to focus on several critical gaps. While the role of gut microbiota in MetS has been extensively explored, further studies are required to elucidate specific microbial species that can serve as therapeutic targets and the mechanisms driving their impact on MetS progression. Similarly, epigenetic regulation remains an underexplored frontier, with potential to uncover novel biomarkers and therapeutic interventions, particularly regarding sex and age-specific epigenetic drift. Finally, while metabolomic profiling has shown promise in identifying early biomarkers for MetS, more large-scale, longitudinal studies are needed to validate these findings across diverse populations and translate them into clinical practice.

## Data Availability

No datasets were generated or analysed during the current study.

## Abbreviations

ACVD: Atherosclerotic Cardiovascular Disease
ADAM: A disintegrin and metalloproteinase
AKT: Protein kinase B
ASCVD: Atherosclerotic Cardiovascular Disease
ATP: Adenosine triphosphate
BMI: Body mass index
cAMP: Cyclic adenosine monophosphate
CD3: Cluster of differentiation 3
CD4: Cluster of differentiation 4
CD8: Cluster of differentiation 8
CD244: Cell surface receptor molecule
CRP: C-reactive protein
CTLA-4: Cytotoxic T-lymphocyte-associated protein 4
CVD: Cardiovascular diseases
ET-1: Endothelin-1
FAA: Free fatty acids
FOXO1: Forkhead box class O-1
FoxP3: Forkhead box P3
GLUT4: Glucose transporter protein
GSK3: Glycogen Synthase Kinase 3
GvHD: Graft-versus-host disease
HCC: Hepatocellular carcinoma
HDL: High-density lipoprotein
HDL-c: High-density lipoprotein cholesterol
IDF: International Diabetic Foundation
IFN-γ: Interferon-gamma
IL-2: Interleukin-2.
IL-6: Interleukin-6
INSR: Insulin receptor
IRS: Insulin receptor
IRS: Insulin Receptor Substrate
JNK: C-Jun N-terminal kinase
LAG3: Lymphocyte-activation gene 3
LDL: Low-density lipoprotein
LDL-c: Low-density lipoprotein cholesterol
MAPK: Mitogen-activated protein kinase
MetS: Metabolic syndrome
mTOR: Mechanistic target of rapamycin
mRNA: Messenger ribonucleic acid
NAFLD: Nonalcoholic fatty liver disease
NO: Nitric oxide
PAI-1: Plasminogen activator inhibitor-1
PD1: Programmed cell death protein 1
PGC1α: Proliferator-activated receptor γ coactivator 1-α
PI3K: Proteins and phosphoinositide 3-kinase
PIP2: Phosphatidylinositol-4,5-bisphosphate
PIP3: Phosphatidylinositol-3,4,5-triphosphate
PKC: Protein kinase Cs
sIL6R: Soluble IL-6 receptor
T2D: Type 2 diabetes
TCR: T-cell receptor
TG: Triglyceride
Th1: Type 1 helper T cells
Th2: Type 2 helper T cells
Th17: Type 17 helper T cells
TIM3: T-cell immunoglobulin and mucin domain containing-3
TNFα: Tumor necrosis factor alpha
TNF-β: Tumor necrosis factor-beta
WC: Waist circumference
VLDL: Low-density lipoprotein
